# High viremia and low level of transmitted drug resistance in anti-retroviral therapy-naïve perinatally-infected children and adolescents with HIV-1 subtype C infection

**DOI:** 10.1186/1471-2334-12-317

**Published:** 2012-11-22

**Authors:** Ujjwal Neogi, Pravat Nalini Sahoo, Ayesha De Costa, Anita Shet

**Affiliations:** 1 Division of Infectious Disease, Department of Medicine, Huddinge, Karolinska Institutet, Stockholm, Sweden; 2 Division of Clinical Virology, Department of Microbiology, St. John’s National Academy of Health Sciences, Bangalore, India; 3 Division of Global Health, Karolinska Institutet, Stockholm, Sweden; 4 Department of Paediatrics, St. John’s National Academy of Health Sciences, Bangalore, 560034, India

**Keywords:** HIV-1 perinatal transmission, Subtype C, Viral dynamics, India

## Abstract

**Background:**

High plasma viremia in HIV-1 infection is associated with rapid CD4 cell decline and faster disease progression. Children with HIV infection have high viral loads, particularly in early childhood. In this study we sought to understand the relationship between duration of HIV-1 infection and viral dynamics among perinatally-infected children and adolescents in India along with transmitted drug resistance in this population.

**Methods:**

During 2007–2011, cross-sectional samples were collected from ART-naïve children (n = 105) with perinatally-acquired HIV infection, aged 2–16 years from Bangalore, India. CD4 counts, viral load and in-house genotyping were performed and transmitted drug resistance mutations were identified using the World Health Organization recommendations for Surveillance of Drug Resistance Mutations (SDRM_2009) list.

**Results:**

Among 105 children studied, 73.3% (77/105) were asymptomatic, but had a median viral load of 5.24 log copies/mL (IQR 4.62-5.66). In the adolescent age group, 54% (21/39) had high levels of viremia (median 5.14 log copies/mL) but were asymptomatic. HIV-1 subtyping identified 98% strains (103/105) as subtype C; one A1 and one unique recombinant form (URF). Transmitted NRTI resistance was 1.9% (2/105); NNRTI resistance was 4.8% (5/105) and overall prevalence of transmitted drug resistance was 5.7% (6/105).

**Conclusions:**

The high burden of plasma viremia found among untreated asymptomatic adolescents needs to be addressed both from an individual angle to halt disease progression, and from a public health perspective to arrest horizontal transmission. The low level of transmitted drug resistance among perinatally-infected children is reassuring; however with improving ART access globally, regular genotyping surveillance is indicated.

## Background

Globally, 3.4 million children below 15 years of age are living with HIV as of 2010 (World Health Organisation, Global summary of the AIDS epidemic, 2010). Among them 1.4 million children reside in the South-east Asia region alone. India has a burden of nearly 2.5 million HIV-1 infected individuals, with children below 15 years of age constituting 3.5% of the total number [1].

Viral load dynamics occurring immediately after infection has been well characterized in adults [2]. Perinatal infection has differing post-transmission dynamics, and consists of a rapid rise in the viral load up to 10^5^ to 10^7^ copies/ml during the first weeks of life followed by a declining trend up to 5 to 6 years [3-5]. Extracellular viral load is a strong indicator of viral replication and high viremia is associated with high transmissibility of HIV-1[6-8]. Although HIV-1 subtype C infection accounts for nearly half (48%) of global HIV infections, few studies have focussed on subtype C viral dynamics. High viremia is seen in a substantial proportion of adults with HIV-1 subtype C infection acquired via horizontal transmission [8,9]. Specific data on viral dynamics among perinatally-infected children are limited [3] and have not been studied within the context of HIV-1 subtype C infection.

Transmitted drug resistance mutation (TDRM) surveillance in children can help to enhance our understanding of transmission of specific clinically-relevant mutations in the era of rapid scale-up of free antiretroviral therapy, particularly prevention of mother-to-child transmission measures. In India, although few studies have previously addressed prevalence of TDRM in adults, [10-12] limited information is available for children and adolescents. The present study aims to understand the relationship between the duration of HIV-1 infection and viremia in children in a setting of HIV-1 subtype C dominance. In addition, the study reports the prevalence of transmitted drug resistant mutations among ART-naive perinatally-infected children.

## Methods

### Study population

The study participants included all children with HIV-1 infection who were regularly visiting the Infectious Disease Clinic at a tertiary care hospital, St. John’s Medical College and Hospital, Bangalore, India since March 2007. Among these, 105 children aged between 2 to 16 years who were perinatally infected, ART-naïve, and with no antenatal exposure to ART, were included in this analysis.

Perinatal infection was confirmed by documented evidence that the mother or both parents were HIV-infected. There was no history of blood transfusion, cross-breast feeding or sexual abuse/exposure among the children included in the study. All the children were therapy-naïve and were diagnosed in early or middle childhood at an average age of 6 years. Maternal HIV status was unknown at the time of birth of these children but was confirmed subsequently, and none of the mothers had received prophylactic antiretroviral drugs during the antenatal period.

### Viral Load, PCR and sequencing

Between 2007 and 2011, a single peripheral blood sample was obtained from each study subject during a routine follow up visit in the clinic. Given that the children in the study were confirmed to be perinatally infected, the duration of HIV infection at the time of sample draw in these children was known. Plasma was separated out after centrifugation and stored at −80 °C until used. Viral load was measured by real time polymerase chain reaction using the Abbott m2000rt system (Abbott Molecular Diagnostics, Germany). Routine CD4 count was measured with Dual-platform flow cytometer (FACS Calibur, BD, USA). PCR and sequencing was carried out as described by us previously [10]. In brief, viral RNA was extracted from plasma using a commercial kit (QIAamp Viral RNA extraction kit, Qiagen, Germany). Partial RT, HXB2 position 2598 to 3250 (corresponds to 17–235 amino acid) regions of the polymerase (pol) gene were amplified using reverse transcriptase polymerase chain reactions (RT-PCR) followed by conventional nested PCR using the following primers, RT04 5^′^-CCTATTGAAACTGTACCAGT-3′ and RT05,5^′^-ACTGTCCATTTATCAGGATG-3^′^ followed by RT07 5^′^AAGCCAGGAATGGATGGCCCA-3^′^ and RT06 5^′^-CCATTTATCAGGATGGAGTTC-3^′^. The purified PCR products were subjected to bidirectional population sequencing using RT06 and RT07 primers in 3730*xl* DNA analyzer (Applied Biosystems, CA, USA) using the second round primers. A consensus sequence was created using BioEdit sequence alignment editor version 7.0.5.3 [13].

### Quality control

An external quality control program by the Quality Control for Molecular Diagnostics, Glasgow, Scotland (QCMD, http://www.qcmd.gov) was maintained by the laboratory where these analyses were performed.

### Reference sequences

Reference sequences (2010) of different subtypes (n = 170) were downloaded from HIV-1 Los Alamos Database (“LANL”, http://www.hiv.lanl.gov). The reverse transcriptase (RT) sequences of previously reported sequences from India, obtained from patients where the therapy status was documented, were downloaded from the same database. A total 595 RT sequences from therapy-naïve patients were downloaded from the LANL database reported in previous studies from India [10-12,14-19]. The downloaded sequences were assessed for accuracy by using the Los Alamos Quality Control tool (LANL_QC) for HIV-1 sequences. Using this tool, sequences with frame shifts, ≥3 stop codons, and APOBEC-mediated hypermutations were excluded, leaving the remaining 567 unique and correctly aligned sequences for the final analysis.

### Phylogenetic analysis, subtyping and recombinant screening

Subtyping was inferred using a maximum likelihood (ML) phylogenetic tree constructed with 1000 bootstrapped data sets, using the Molecular Evolutionary Genetics Analysis software version 5 (MEGA 5) with the subtype reference sequences downloaded from the database [20]. All sequences were further submitted to Recombination Identification Program 3.0 (RIP 3.0). The sequences which showed recombination were further processed for detailed analysis of recombination breakpoints. Recombination patterns were determined by performing bootscan analysis with Simplot version 3.5.1 [21], using a window sliding of 100 nucleotide (nt) in 10nt steps, with 500 bootstrap replicates. The mosaic pattern of each URF was confirmed by phylogenetic analysis of the recombination fragments using the same parameters as described above.

### Drug resistance genotyping and definition of transmitted drug resistance mutations

Genotyping was performed on plasma samples derived from ART-naïve children. Briefly, the reverse transcriptase (RT) region of HIV-1 *pol* gene was amplified and sequenced using the primers described above. These sequences were analysed for the presence of transmitted drug resistance using the World Health Organization (WHO) recommendations for surveillance of drug resistance mutations updated in 2009 (SDRM_2009) [22]. This list includes 34 NRTI-resistance mutations at 15 RT positions, and 19 NNRTI-resistance mutations at 10 RT positions [22]. We also used the International AIDS Society-USA updated drug resistance mutations in HIV-1 (IAS-USA 2011) for interpretation of TDRM. IAS-USA 2011 includes 19 NRTI mutations in 16 positions, and 34 NNRTI mutations in 16 positions between amino acid residues 17 to 235 of RT region of *pol*[23]. To further increase the accuracy of this analyses, we eliminated certain mutations included in the SDRM_2009 or IAS Panel, which displayed characteristics of polymorphisms (defined as those mutations which are normally present at a frequency of ≥0.5% in therapy-naïve patients, and at a frequency of <0.5% among those failing therapy) [22].

### Intra-population divergence

The genetic distance of each of the study sequences to the Indian consensus C sequence (intra-population divergence) was calculated in MEGA 5 software [20].

### Statistical methods

Descriptive statistics like mean, median were calculated in SPSS ver 16.0. A spearman rank co-relation (r_s_) was used for analysis of potential co-relation between continuous variables. *P* value <0.05 was considered as significant.

### Ethical statement

The study has been approved by institutional ethical review board, St. John’s Medical College Hospital, Bangalore, India. Written informed consent was obtained from all caregivers prior to recruitment of the children, and a verbal assent was obtained from children > 8 years of age.

## Results

### Study population characteristics

Among the total of 105 children studied, mean age was 8 years (SD ± 3.4) and median CD4 count was 530 cells/mm^3^ (IQR 338, 780) (Table 1). Median viral load was 5.24 log copies/mL (IQR 4.62, 5.66) and 73.3% of these children (77/105) were classified as clinically stable (WHO clinical stage I or II) and remaining 28 children were classified as symptomatic (WHO clinical stage III or IV). As perinatal infection was the most likely mode of transmission, the duration of infection was deemed to be equivalent to the age of each child.

**Table 1:** Patient demography and clinical status of the study participants

	**Over all (n = 105)**	**<5 years (n = 17)**	**≥5 to <10 years (n = 49)**	**≥10 years (n = 39)**
Sex: Male (%)	61 (58.1%)	12 (70%)	29 (59%)	20(51%)
CD4-T cell count	530 (338–780)	1051 (528–1154)	586 (401–768)	373 (251–578)
Median (IQR*)				
Viral load log copies/mL	5.24 (4.62-5.66)	5.56 (5.51-6.05)	5.12 (4.55-5.55)	5.2 (4.73-5.63)
Median (IQR*)				
WHO Clinical stage				
Stage 1	40 (38.1%)	6 (35.3%)	19 (38.7%)	15 (38.5%)
Stage 2	37 (35.2%)	5 (29.4%)	19 (38.7%)	13 (33.3%)
Stage 3	22 (21%)	4 (23.5%)	9 (18.4%)	9 (23.1%)
Stage 4	6 (5.7%)	2 (11.8%)	02 (4.1%)	2 (5.1%)

*IQR: Inter-quartile range.

### Extended high viremia and CD4 count

**Figure 1:**
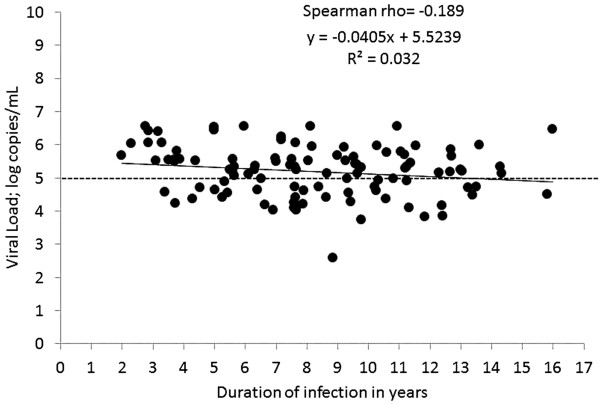
**Association between duration of infection and log transformed viral load.** The graph represents association between log transformed viral load and length of the infection in perinatal transmission. No significant correlation between the parameters observed (Spearman rho = −0.189; p = 0.05).

Viral load values remained similar regardless of age, and showed no significant correlation with duration of infection in these populations (Figure 1). CD4 T-cell count weakly correlated with viral load among children ≥5 years of age (n = 88; Spearman rho = −0.26). Among these 88 children, 58% (51/88) were extended high viremics (>5.0 log copies/mL as described previously [8]) and among these, 67% (34/51) had CD4 count higher than 350 cells/mm^3^. In the adolescent age group (10–16 years of age, n = 39), 54% (21/39) had extended high viremia (median 5.14 log copies/mL; IQR 4.69-5.34) despite having a CD4 T-cell counts >350 cells/mm^3^.

### HIV-1 subtyping

**Figure 2:**
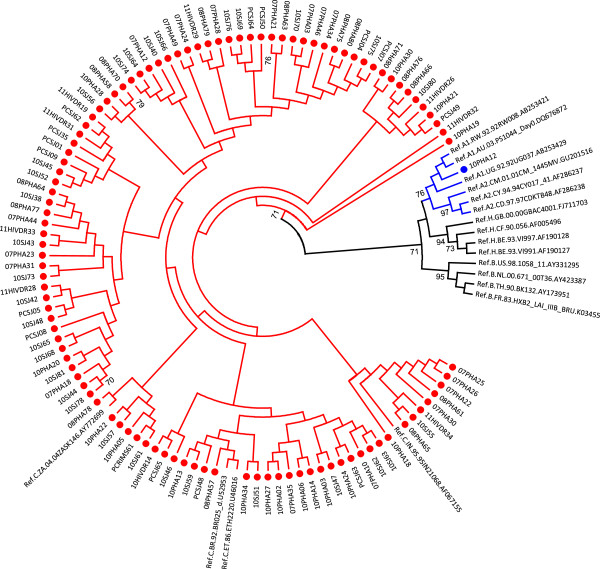
**Identification and characterisation of HIV-1 subtypes.** The tree was constructed with cohort sequences and reference sequences downloaded from Los Alamos Database (http://www.hiv.lanl.gov) using general time reversible (GTR) model with gamma distribution and invariant sites (GTR + G + I) as observed best fitted model for the dataset. Cohort sequences are shown with filled circle.

**Figure 3:**
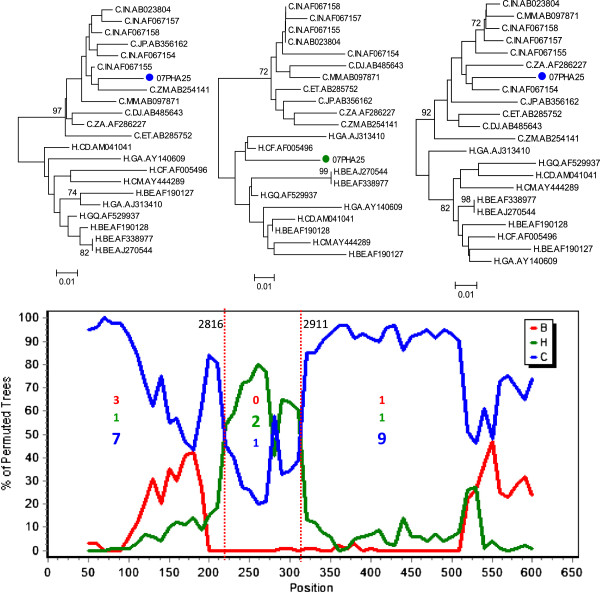
**Mosaic structure of identified unique recombinant forms (URF) strains.** The strain, which was determined as URF_CH in RIP 3.0 program was further studied in details for its mosaic structure. The recombination patterns were determined by performing bootscan analysis with Simplot version 3.5.1 [21], using a window sliding of 100 nucleotide (nt) in 10nt steps, with 500 bootstrap replicates. The informative sites were presented inside the graph. The mosaic pattern of each segments were confirmed by phylogenetic analysis using reference subtype C and H strains downloaded from Los Alamos Database (http://www.hiv.lanl.gov). Two breakpoints were identified at 2816 and 2911 of HXB2 co-ordinates.

HIV-1 subtyping based on RT region of *pol* gene (HXB2 position 2598 to 3250) identified 98% strains (103/105) as subtype C which is predominant in India [24] (Figure 2). One sample was identified as subtype A1 and one as a unique recombinant form CH (URF_CH). (Figure 3).

### Transmitted drug resistance and polymorphisms

Using the SDRM_2009 list [22] for drug resistance monitoring, we found that among the 105 ART-naïve children, 3.8% (4/105) had transmitted NRTI drug resistance mutations (L74I, T69D, T215S and K219Q) and 4.8% (5/105) had NNRTI drug resistance mutations [K103N (n = 2), K101E (n = 2), Y181C (n = 1)]. After excluding likely polymorphisms such as L74I and T215S, transmitted NRTI resistance prevalence was 1.9% (2/105). The overall prevalence of transmitted drug resistance in this population was 5.7% (6/105). Among the six strains, only one had two classes of mutations (both NRTI and NNRTI) (Table 2).

**Table 2:** Profile of children with transmitted drug resistance

**Patient No**	**Length of infection (years)**	**WHO Stage**	**CD4 Count (cells/mm**^**3**^**)**	**Viral Load (log copies/mL)**	**NRTI SDRMs**	**NNRTI SDRMs**
1	16	3	221	6.46	None	K103N
2	11	3	417	5.34	None	K101E
3	11	4	14	5.69	T69D	None
4	9	1	247	5.08	None	K103N
5	9	1	401	5.50	K219Q	K101E
6	6	2	288	6.55	None	Y181C

When the IAS-USA 2011 list [23] was used for TDRM interpretation, a slightly differing result was obtained. The NRTI resistance prevalence was 0.9% (1/105; K219Q) and NNRTI resistance was 5.7% [6/105; K103N (n = 2), K101E (n = 2), Y181C (n = 1), F227C (n = 1)]. But the overall transmitted drug resistance prevalence remained the same; 5.7% (6/105).

The profiles of children with transmitted drug resistance are presented in Table 2. Children who had TDRM (n = 6), had higher mean viral load compared to those with no TDRM (n = 99) (5.75 vs 5.16 log copies/mL; p = 0.06) although only a trend towards significance was seen due to the small sample size. Among the six children who had TDRM, 3 children had severe immunosuppression and were deemed eligible to start ART.

## Discussion

Our study on viral dynamics and drug resistance testing among ART-naive perinatally-infected children aged between 2 and 16 years found that a substantial proportion of older children with CD4 T cell count in the ‘non-immunosuppressed’ category had high viremia. As expected subtype C is the most prevalent HIV-1 subtype in this study population. We also identified a low level of transmitted drug resistance in this population.

A recent study showed that a proportion of HIV-infected adults with horizontally-acquired subtype C infection in southern Africa maintained high viral loads for a prolonged period of time [8]. A South African cohort of HIV-1-infected infants also demonstrated high viral load where HIV-1 subtype C is predominant [25]. A comparative study between Malawi and US/Swiss study participants also showed a significant higher viral load in Malawians, suggesting the possibility that high viremia may occur more frequently in subtype C rather than subtype B infection [9]. In our cohort, the duration of infection as measured by the age of these children, did not directly correlate with the degree of viremia, thus precluding viral load measurement as a prognostic marker of disease length; moreover, there was a substantial proportion of adolescents who maintained high CD4 T-cell count but had elevated viremia as well. Our findings imply that even in vertical transmission, a proportion of HIV-1 subtype C-infected children and adolescents have high viremia irrespective of their stable clinical and immunological status. To minimize horizontal transmission among youth, targeted interventions with treatment as prevention among adolescents with high viremia may be of public health benefit which could also be a cost-effective strategy to attain maximum population-level reductions in HIV-1 transmission [26].

The transmitted drug resistance prevalence was derived by using the SDRM 2009 list [22]. The SDRM 2009 list was compiled keeping in mind four main concepts: (i) included mutations should be recognized as causing or contributing to drug resistance - by being present on ≥3 of five existing expert lists of drug resistance mutations, namely, Stanford university HIVdb drug resistance interpretation algorithm (http://hivdb.stanford.edu/). IAS-USA Mutations Associated With Drug Resistance (March/April 2008), ANRS drug resistance interpretation algorithm, Los Alamos National Laboratories HIV Sequence database (2007), or Rega Institute Drug Resistance Interpretation Algorithm (7.1.1) (ii) the mutations should not be polymorphisms or occur at highly polymorphic positions, (iii) the mutations should be applicable to the 8 most common HIV-1 subtypes globally and (iv) the list should be parsimonious, excluding mutations that are unlikely to result from drug pressure. These criteria have shaped the SDRM 2009 list to be especially relevant in our setting, and hence this list was used for our current interpretation. The TDRM level in our study population was very low which was consistent with our previous findings where the level of TDRMS and polymorphisms among ART-naïve adults did not exceed 10% [10]. Other paediatric studies from India have revealed similar low TDRM prevalence; among a south Indian cohort of 48 perinatally infected children of mean age 5.7 years, none had major drug resistance mutations [27]. In another study from north India where 25 ART-naïve children with mean age 6 years were studied, K103N was the only drug resistance mutation that was observed [28]. Studies on ART-naïve adults from different parts of India with sample sizes ranging from 34 to 107 patients, have revealed low TDRM prevalence (<5%) [11,12,14-18].

Presence of K103N and K101E TDRM in our cohort sequences may be due to wide usage of nevirapine in this setting [29]. Our data on prevalence of TDRM among children was significantly lower than other settings. Studies from Brazil reported 26.9% of TDRM in therapy-naïve children [30], while a report from Central African Republic identified 13.9% [31]. A meta-analysis showed that approximately half of the children with PMTCT exposure (single-dose nevirapine) harboured TDRM [32]. A recent study from Spain on PMTCT-exposed children identified 13% TDR [33], while another study from Western Cape Province of South Africa reported TDRM <5% among children who were exposed to PMTCT [34]. A recent study also showed that the effect of viral fitness on HIV RNA level could be one of the determining factors for selective viral transmission; maternal presence of mutations such as K103N which promote viral fitness may be easily transmitted to infants [35].

Minor differences between the two lists used for TDRM interpretation can lead to contradictory prevalence results of drug resistance. In our study, we excluded minor NRTI mutations such as T215S and L74I which were included in the SDRM 2009 list. The SDRM 2009 WHO group recommends that the SDRM list should be “as parsimonious as possible without sacrificing sensitivity,” this can be accomplished by excluding “exceedingly rare drug-resistance mutations defined as those mutations present at a frequency below 0.5% among treated individuals in the subtype having the highest prevalence of that mutation” [22]. Our analysis of previous RT sequences obtained from treatment-experienced patients from Indian subtype C patients [n = 606; downloaded from the Los Alamos database (n = 102); literature survey (n = 446) and unpublished from lab (n = 58)] revealed that T215S was not present in any of the sequences (data not shown). Although *in vitro* analysis showed that T215S represents revertants of T215Y [36], phenotypic characterisation did not show resistance to any nucleoside analogues including zidovudine [37]. The L74I mutation was present in 0.3% (2/606) of sequences obtained from treatment-experienced patients infected with subtype C viruses. In addition, the analysis of global subtype C sequences in HIVseq program available in Stanford University HIV drug resistance database (http://hivdb.stanford.edu/; accessed 07 October 2012) also revealed absence of both mutations in 232 HIV-1 subtype C sequences from NRTI-treated patients. The low prevalence of T215S and L74I at a frequency of <0.5% in treatment-experienced patients harbouring subtype C HIV-1 provided the basis for our rationale for excluding these mutations from our analysis. F227C is another example of a mutation included in the IAS-USA panel that is not likely to be significant as a *transmitted* mutation, as F227C confers “minor” resistance to rilpivirine, a drug that is as yet unavailable in India. Other studies have observed differences in prevalence of TDRM within the same cohort when differing tools such as SDRM 2009 and IAS-USA 2009 are used [38]. Current SDRM 2009 guidelines propose that such polymorphic drug resistance mutations which occur commonly in the absence of drug selective pressure, and are present in <0.5% of the treated population, could lead to falsely elevated estimates of TDRM, and hence, must not be included for surveillance of transmitted HIVDR [22].

Our study limitations include the cross-sectional design which restricts our ability to comment on kinetics of viral dynamics and evolution. Further, we have not been able to include samples from children below two years of age for this analysis as most of the children in our settings were diagnosed in their early and middle childhood. There may be an inherent survival bias since only these older children were included in the study. Thus, having only chronically infected children may actually underestimate the prevalence of transmitted drug resistance as the wild type virus with increased fitness may predominate and minor viral populations with drug resistant mutations may be too low to detect using population sequencing. However, our study is strengthened by the inclusion of a high proportion of children with age ≥10 years, which allowed us to study viral load and its associations in adolescents. Finally, the population-based genotyping method can only detect mutations that are present in 30% or more of the quasi-species constituting circulating virus population [39]. It is possible that mutations carried by minor viral populations may be missed due to the limited sensitivity of population-based sequencing method.

## Conclusions

Our study is the first of its kind which describes the relationship between the length of infection and the degree of viremia in perinatally-infected children and adolescents living with HIV-1 subtype C in India. A significant finding of this study is the inconsistent clinical parameters of high viral load and high CD4 count co-existing in over half the adolescents studied, where the duration of infection is >10 years. Studies have shown a high probability of disease progression to AIDS among those with high viremia [40]. Will this group of adolescent extended high viremics experience imminent rapid disease progression as has been seen in adult studies? It is conceivable that extended high viremia with drug resistance mutations in untreated adolescents may also play a role in fuelling the HIV epidemic. Therefore, early initiations of ART while emphasizing good adherence may be considered in this population in order to attain maximum population-level reductions in HIV-1 transmission. The evidence from this study also suggests that the interpretation of prevalence of TDRM in population-based surveillance should be used with caution to avoid reporting falsely elevated TDR prevalence in the population. The low level of transmitted drug resistance in children unexposed to antenatal antiretroviral drugs, although reassuring, should not advocate complacency. Regular surveillance is necessary to understand the evolution of drug resistance mutations, particularly with increasing PMTCT coverage nationally and globally.

## Competing interests

The authors declare that they have no competing interests.

## Authors’ contributions

UN conceived of the study, and participated in its design, carried out the drug resistance genotyping, bioinformatics analysis, performed the statistical analysis and drafted the manuscript. PNS carried out the lab works, helped in clinical data accumulations. ADC performed the statistical analysis, inputs in the public health aspects and critically reviewed the manuscript. AS conceived of the study, and participated in its design and critically reviewed the manuscript. All authors read and approved the final manuscript.

## Pre-publication history

The pre-publication history for this paper can be accessed here:

http://www.biomedcentral.com/1471-2334/12/317/prepub
